# The impact of COVID-19 on pragmatic clinical trials: lessons learned from the NIH Health Care Systems Research Collaboratory

**DOI:** 10.1186/s13063-022-06385-8

**Published:** 2022-05-21

**Authors:** Emily C. O’Brien, Jeremy Sugarman, Kevin P. Weinfurt, Eric B. Larson, Patrick J. Heagerty, Adrian F. Hernandez, Lesley H. Curtis

**Affiliations:** 1 Department of Population Health Sciences, School of Medicine, Duke University, 215 Morris Street, Suite 210, Durham, NC 27701 USA; 2 Duke Clinical Research Institute, School of Medicine, Duke University, Durham, NC USA; 3 Berman Institute of Bioethics, Johns Hopkins University, Baltimore, MD USA; 4 Kaiser Permanente Washington Health Research Institute, Seattle, WA USA; 5 Department of Biostatistics, University of Washington, Seattle, WA USA; 6 Department of Medicine, School of Medicine, Duke University, Durham, NC USA

**Keywords:** Pragmatic clinical trials, Research participation, COVID-19

## Abstract

**Background:**

The COVID-19 pandemic has considerably disrupted nearly all aspects of daily life, including healthcare delivery and clinical research. Because pragmatic clinical trials are often embedded within healthcare delivery systems, they may be at high risk of disruption due to the dual impacts on the conduct of both care and research.

**Methods:**

We collected qualitative data using multiple methods to characterize the impact of COVID-19 on the research activities of 14 active pragmatic clinical trials in the National Institutes of Health (NIH) Health Care Systems Research Collaboratory. A COVID-19 impact questionnaire was administered electronically to principal investigators in June 2020. Text responses were analyzed thematically, and qualitative summaries were subsequently reviewed by five independent reviewers, who made iterative revisions. Additional COVID-19-related impacts were identified during virtual meetings with trial teams during April–July 2020 and combined with questionnaire responses for analysis.

**Results:**

Impacts of the pandemic were broadly classified into two main types: healthcare operations and social distancing. In some instances, trial delays created statistical challenges, particularly with trials using stepped-wedge designs, and necessitated changing data collection strategies or modifying interventions. The majority of projects used existing stakeholder-driven approaches to adapt interventions. Several benefits of these adaptions were identified, including expanded outreach capabilities and ability to study virtual intervention delivery. All trial teams were able to adapt to pandemic-related modifications.

**Conclusion:**

In a group of 14 ongoing pragmatic clinical trials, there was significant impact of COVID-19 on trial activities. Engaging appropriate stakeholders was critical to designing and implementing trial modifications and making continued safe progress toward meeting research objectives.

**Supplementary Information:**

The online version contains supplementary material available at 10.1186/s13063-022-06385-8.

## Background

Since the onset of the COVID-19 pandemic in early 2020, there have been substantial disruptions to virtually all aspects of daily life, including clinical care and research [1]. Many ongoing clinical trials and prospective studies were paused, research efforts were redirected to COVID-19, and myriad clinician scientists were redeployed to delivering care [2, 3]. In addition, in effort to reduce viral transmission, most healthcare systems temporarily reduced or eliminated non-essential patient visits [4, 5]. A growing body of evidence demonstrates major changes in care-seeking behavior during the pandemic, including for emergency conditions [6, 7]. These disruptions in usual care and behavior complicate research embedded within healthcare systems and may result in both analytic and operational challenges for this research. However, because of their streamlined procedures and emphasis on use of existing data [8], pragmatic clinical trials may also offer novel ways of adapting to major disruptions, such as those resulting from the COVID-19 pandemic.

The National Institutes of Health (NIH) Health Care Systems Research Collaboratory (the “Collaboratory”) involves an infrastructure to conduct collaborative research across healthcare systems (https://rethinkingclinicaltrials.org/). The Collaboratory consists of five Cores (Biostatistics and Study Design, Electronic Health Records, Ethics and Regulatory, Health Care Systems Interactions, and Patient-Centered Outcomes) and a Coordinating Center, which work to produce, document, and disseminate standards for healthcare system-based pragmatic research [9]. Collaboratory Cores support demonstration projects in a number of ways, including advising on study design and statistical analysis, facilitating interactions with health system leadership and operations relevant to the trial, and providing feedback on dissemination and implementation strategies, among others. To date, the Collaboratory has supported the design and conduct of 21 demonstration projects, large-scale pragmatic clinical trials on a range of conditions that engage healthcare delivery organizations as research partners (https://rethinkingclinicaltrials.org/demonstration-projects).

In this paper, we report the experience of the Collaboratory during the early COVID-19 pandemic. Specifically, we review the results from a survey of the pragmatic trial teams aimed at understanding the specific ways in which they were affected by the pandemic. We supplement these survey data with information obtained during communications between the Collaboratory Coordinating Center and project teams. As such, we describe not only the general effects of COVID-19 on the projects themselves, but also the adaptations studies made to data collection strategies and modifications to their interventions and/or implementation. These collective experiences enable us to offer considerations for describing, measuring, analyzing, and reporting changes to study domains as a result of the pandemic, which may potentially be useful in the event of other major future disruptions.

## Methods

A COVID-19 impact questionnaire was developed by the Collaboratory Coordinating Center in consultation with NIH program leadership. Using a previously developed questionnaire from the NIH-DoD-VA Pain Management Collaboratory [1], the Collaboratory Coordinating Center team members adapted survey questions to align with the goal of characterizing the impact of the pandemic on Collaboratory pragmatic trials. Survey themes of interest ranged from broad (e.g., “How has COVID-19 impacted your trial?”) to specific (e.g., “Which key individuals or stakeholders have you engaged due to COVID-19?”). Survey questions were structured as open-ended to allow capture of a broad range of possible impacts. The final questionnaire is included in Supplementary Materials. The survey was considered to be part of the Coordinating Center project objectives of collecting generalizable information and creating best practices for pragmatic research. These Coordinating Center activities were determined to be exempt from IRB review by Duke University Health System IRB (protocol ID: Pro00085360). The survey was fielded with all active Collaboratory demonstration projects (*n*=14) (Table 1). Specifically, it was administered in June 2020 by email to the trial principal investigators.

**Table 1:** Active Collaboratory demonstration projects at the outset of the pandemic

Acronym (ClinicalTrials.gov identifier)	Trial name	Therapeutic area
*Implementation phase*		
ACP PEACE (NCT03609177)	Improving Advance Care Planning: Promoting Effective and Aligned Communication in the Elderly	Cancer
EMBED (NCT03658642)	Pragmatic Trial of User-Centered Clinical Decision Support to Implement Emergency Department-Initiated Buprenorphine for Opioid Use Disorder	Opioid use disorder
GGC4H (NCT04040153)	Guiding Good Choices for Health: Testing Feasibility and Effectiveness of Universal Parent-Focused Prevention in Three Healthcare Systems	Adolescent behavioral health
HiLo (NCT04095039)	Pragmatic Trial of Higher vs. Lower Serum Phosphate Targets in Patients Undergoing Hemodialysis	Hemodialysis
ICD-Pieces (NCT02587936)	Improving Chronic Disease Management with Pieces	Chronic kidney disease, diabetes and hypertension
Nudge (NCT03973931)	Personalized Patient Data and Behavioral Nudges to Improve Adherence to Chronic Cardiovascular Medications	Cardiovascular disease
PRIM-ER (NCT03424109)	Primary Palliative Care for Emergency Medicine	Palliative care
*Pilot phase*		
BackInAction (NCT04982315)	Pragmatic Trial of Acupuncture for Chronic Low Back Pain in Older Adults	Chronic pain
FM-TIPS (NCT04683042)	Fibromyalgia TENS in Physical Therapy Study	Fibromyalgia/chronic pain
NOHARM (NCT04570371)	Nonpharmacologic Options in Postoperative Hospital-based and Rehabilitation Pain Management	Post-surgical pain
OPTIMUM (NCT04129450)	Group-based Mindfulness for Patients with Chronic Low Back Pain in the Primary Care Setting	Chronic pain
*Completed enrollment*		
PROVEN (NCT02612688)	Pragmatic Trial of Video Education in Nursing Homes	Advanced care planning
SPOT (NCT02326883)	Suicide Prevention Outreach Trial	Behavioral health
TSOS (NCT02655354)	A Policy-Relevant U.S. Trauma Care System Pragmatic Trial for PTSD and Comorbidity (Trauma Survivors Outcomes and Support)	Behavioral health

During the COVID-19 pandemic, Cores consulted with project teams on regular virtual meetings as well as upon request. Project teams also provide periodic updates to the Collaboratory Steering Committee, which consists of Coordinating Center principal investigators, Core leaders, trial principal investigators, and NIH project leadership. Given the direct relevance of discussions during Core and Steering Committee virtual meetings to measurement of the pandemic’s impact on trial conduct, we compiled information from these discussions into a database and combined them with survey data for analysis. All 14 project teams either completed the COVID-19 questionnaire and/or supplied information related to the effects of the COVID-19 pandemic on their project activities during a telephone consultation or a virtual Steering Committee meeting. Seven were conducting a full-scale pragmatic clinical trial and four were in the pilot phase when the COVID-19 pandemic began. Three trials had completed enrollment at that time.

Given the open-ended nature of the prompts, all data collected in the survey and group discussions were qualitative in nature, and no statistical analysis was performed. Text from the survey responses and virtual meeting/consultation notes was analyzed thematically by a single reviewer, who wrote descriptive summaries for each survey question. The thematic analysis was subsequently reviewed by five additional independent reviewers, who made updates to the descriptive summaries via an iterative email-based process to incorporate revisions to the written analytic summary.

## Results

We describe two main types of impacts of COVID-19 on demonstration projects: healthcare operations and social distancing. Then, we describe specific changes to data collection strategies and modifications to the intervention and/or implementation as a result of the pandemic, as well as any benefits that study teams perceived to be associated with these changes. Finally, we describe planning and next steps for the projects.

### Healthcare operations

While delays were common in most types of research in spring 2020, the impact of COVID-19 on pragmatic trials was complicated by the pandemic’s overarching effects on the health systems in which they were embedded. System-wide research pauses resulted in delays in conducting in-person trainings and other site activation activities for at least two projects. Investigators of four trials also reported system-level delays in contracting/reliance agreements and the temporary suspension in delivery of some healthcare services. The FM-TIPS trial experienced changes in Institutional Review Board requirements for initiation of research with human subjects.

Six trials reported system-level efforts to prioritize COVID-19-related research and limit nonessential care, which affected staff availability and delayed trial operations. Four projects reported adverse impacts of staffing changes, including reallocation of staff to COVID-19-related research. In FM-TIPS, physical therapy staff had their hours reduced or were furloughed. In BackInAction, recruitment of new staff was delayed for 3 months. Staffing changes due to COVID-19 were so significant in the HiLo trial that the investigative team anticipated the study remaining on hold until the effects of the pandemic on the sites were eased. For staff continuing trial-related work, changing clinic requirements with respect to use of personal protective equipment, cleaning procedures, and telehealth delivery introduced general disruption and additional time constraints as clinic staff adapted to this new environment.

Four trials reported minimal impacts of the pandemic due to pre-existing features of the study design and/or the target population. The BackInAction trial team anticipated few significant impacts due to COVID-19, as diagnoses of the target condition were likely to remain high enough despite potential disruptions in primary care services. In general, trials least affected by healthcare operations-related disruptions were those with enrollment systems already in place and those relying heavily on automated data collection through the electronic health record and/or mobile technologies.

### Social distancing

Many projects reported having large numbers of staff members transitioning to working remotely and using virtual communication methods to continue their trials. Transitions to remote work commonly resulted in delays as research staff adapted to virtual project operations. Project teams reported that changing to virtual intervention delivery often required technology training, and in one project, a staff person assigned for technology assistance. The PRIM-ER trial stated that system-wide travel restrictions and group assembly rules might affect trial-related training sessions. Finally, transition to remote work impacted timelines of project vendors, which resulted in delays due to vendor production of one trial’s intervention device.

Social distancing also altered participation. Several projects reported fewer provider referrals to specialty care and lower patient attendance at referral appointments, which impacted recruitment. Investigators from the BackInAction trial reported observing that patients with pain were experiencing exacerbations due to social distancing, and the delivery of in-person pain treatments like acupuncture was more appealing to patients when such procedures could be done safely.

### Project changes in response to COVID-19

#### Stakeholder consultation

Many project teams needed to adapt data collection strategies, modify interventions, or alter implementation plans. The process involved the input of stakeholders. That is, a variety of stakeholders were consulted or involved in assessing the potential impact of COVID-19 and determining appropriate solutions for each trial (Table 2). In most cases, the stakeholders consulted had a pre-existing relationship with the trial and were consulted for this new reason. In a few cases, new stakeholders were consulted, primarily those who provided guidance with respect to COVID-19 impacts on practice within the health system and virtual intervention delivery.

**Table 2:** Stakeholders supporting demonstration project intervention adaptation in response to COVID-19

Funding agency	Collaboratory Coordinating Center	Health systems	Trial team
∙ Funding agency leadership ∙NIH Program Officer	∙ Biostatistics and Study Design Core ∙ Coordinating Center ∙ Electronic Health Records Core ∙ Ethics and Regulatory Core ∙ Health Care Systems Interactions Core	∙ Health system managers, clinic liaisons, site healthcare providers, and site support staff ∙ Information Technology leadership ∙ Non-study team members from the research institutions ∙ Nursing leadership ∙ Practice leadership ∙ Provider organization leadership	∙ Biostatisticians ∙ Data and safety monitoring board ∙ Intervention experts ∙ Trial stakeholder panel ∙ Trial steering committee and executive committee members

The Collaboratory Coordinating Center and its Cores supported the project teams in several specific ways through regularly scheduled virtual meetings and ad hoc consultations. The Biostatistics and Study Design Core worked with each project to help identify COVID-19’s impacts, make any necessary changes to the study designs or statistical analysis plans, and collaboratively ascertain cross-study guidelines that could facilitate efficient responses to these emerging issues. The Electronic Health Records Core collected details on whether project teams were making changes to the information they collect to account for the potential impacts of the pandemic. The Core also shared updates from major standards development organizations on the availability of diagnostic and procedure codes related to COVID-19. The Health Care Systems Interactions Core and project teams discussed the impact of COVID-19 on relationships with health system partners, and the Core helped to identify and navigate issues with sites and staffing. The Ethics and Regulatory Core worked with project teams on issues related to virtual healthcare delivery, such as privacy, accessibility, and permissibility across jurisdictional boundaries.

#### Implementation of changes

Following these consultations, trial teams implemented a number of solutions to COVID-19-related research challenges. The most common solution implemented across all projects was a shift from in-person interactions to virtual, whether for team communication or patient interaction. In the case of intervention delivery, this shift required careful consideration as to whether the intervention would be as effective when delivered virtually. Contingency planning for possible delays was also described. In the NOHARM trial, investigators noted, “we adjusted the scale of our pilot and revised our milestones commensurate with practice timelines and travel restrictions.” Among the trials adapting to conduct some or all of their intervention virtually, GGC4H anticipates a permanent change to virtual delivery for consistency over the duration of the trial. ACP PEACE continued to deliver their intervention virtually due to travel restrictions and several observed advantages of virtual delivery, including enhancements to learning and easier participation for oncologists. HiLo specifically excluded clinics with separate units for COVID-19-positive patients and, as a result, increased the number of clusters to maintain study power. In the Nudge trial, which used prescription refill text reminders, trial investigators were concerned that patients may pick up medications in-person, resulting in increased risk of COVID-19 exposure for their high-risk population, so they modified their text messages to encourage patients to refill medications via mail or have family members who were at lower risk for COVID-19 pick up their prescriptions.

Trial teams also reported several changes to planned data collection. Two trials considered whether to collect data related to COVID-19 in addition to originally planned data elements. The BackInAction team weighed increased participant burden as a result of adding COVID-19 measures, but ultimately added questions about COVID-19 to the outcome measures collected as part of the trial. They used focus groups and interviews with participants that were already underway during their pilot phase, but converted to virtual format, to ask about any new factors that may affect patients’ decisions to participate in this kind of trial given the context of COVID-19-related health risks.

When queried about changes to implementation plans, several trial teams reported modifying the enrollment schedule with a slower startup for clinic site enrollment, postponing site activation for sites anticipating severe impacts from COVID-19, and conducting virtual focus groups/qualitative interviews to better understand patients’ interest in the virtual intervention delivery. The GGC4H trial conducted virtual focus groups to ascertain parental interest in the intervention amidst the pressures of the pandemic and called intervention participants who missed a group session to encourage them to attend the next session.

#### Statistical challenges

Investigators faced several statistical challenges due to COVID-19-related trial delays, particularly with stepped-wedge designs where interventions were rolled out to sites in a staggered fashion. In the GGC4H trial, these delays also created an extended gap between baseline assessment and intervention delivery, requiring the addition of a second baseline assessment. Several project teams recognized that their outcomes of interest could be affected by COVID-19; for example, some anticipated increases in rates of hospitalization and death as well as missed ongoing care visits, resulting in missing follow-up data. In the ACP PEACE trial, which is studying advance care planning (ACP), there was an apparent increase in ACP activities in response to the disproportionate risk from COVID-19 and higher mortality rates among older patients in both control and intervention clinics. As a result of this modification to the baseline rate of ACP, the project team decided to use the original step 2 period of the stepped-wedge trial as its new baseline, in effect restarting the trial.

Statistical approaches were employed widely to determine the potential effects of COVID-19 on the trials and to make necessary adjustments in formal analysis plans in consultation with the Collaboratory Biostatistics and Study Design Core. For example, the HiLo trial will be conducting simulations to determine the potential impact of COVID-19-related events on the primary outcome particularly given the potential impact of the pandemic on infections within dialysis units (trial clusters). The ICD-Pieces trial will be monitoring the effects of COVID-19 on missing data to see if any alternative methods of data collection are needed. The Nudge trial had discussions about effects of COVID-19 on its outcomes of death and hospitalization, but determined changes to the study were not needed as the effects should be evenly distributed due to the randomly assigned treatment arms.

#### Considerations for impact analysis

Given that multiple trial domains were impacted by COVID-19-related changes, the process for understanding and/or quantifying these impacts may differ depending on the domain(s) affected. The Biostatistics and Study Design Core summarized considerations for measurement and analysis of COVID-19-related impacts by domain based on their consultations with trial teams (Table 3). These considerations are described within each of the six PICOTS framework domains (Population, Intervention, Comparison, Outcome, Timing, and Setting) and are broadly organized by those related to documentation and measurement, and those related to analysis and reporting.

**Table 3:** Considerations for measurement and analysis of COVID-19 impacts by PICOTS domain

PICOTS Clinical research element	Document/measure	Analysis/report
Patient population	∙ Measure changes to participant demographic and clinical characteristics ∙ Measure changes to access/attitudes (i.e., Coronavirus Impact Scale) ∙ Evaluate COVID-related methods for electronic phenotyping	∙ Compare characteristics over time periods (i.e., pre and post March 2020) ∙ Compare characteristics over time periods defined by local COVID status or impacts on research conduct
Intervention of interest	∙ Document modifications to ensure the safety of patients and providers ∙ Document/measure changes to the methods to communicate and deliver intervention ∙ Document/measure changes to any components or mechanisms associated with intervention	∙ Compare engagement in intervention components over time periods ∙ Consider modification to primary analysis to adjust (stratify) for COVID phases ∙ Consider sensitivity analyses that restrict to periods of time based on COVID status ∙ Consider subgroup (interaction) analysis of heterogeneity of treatment effect (HTE) across COVID time periods ∙ Consider time-specific mediation analysis to evaluate putative mechanisms across COVID time periods
Comparison intervention	∙ Document modifications to ensure the safety of patients and providers ∙ Measure trends in concomitant care over time	∙ Compare engagement in comparison (usual care) over time periods ∙ Clarify the estimand of interest—typically the average treatment effect that averages over explicit patient and time characteristics while potentially controlling certain factors ∙ Consider subgroup (interaction) analysis of HTE across COVID time periods ∙ Report the consequence of COVID impacts that are specific to the study design (i.e., parallel randomized trials will be impacted differently from crossover or stepped-wedge designs)
Outcome(s)	∙ Document/measure changes in the way that clinical assessments and patient-reported outcomes are obtained ∙ Measure the impact of COVID-related distress on patient-reported outcomes	∙ Report the psychometric properties of any modified measures ∙ Consider analysis of subgroups based on patient-reported characteristics at baseline that are predictive of outcomes or COVID susceptibility ∙ Describe temporal trends in patient-reported outcomes ∙ Evaluate patterns and correlates of missing data over time to assess differential non-response
Timing	∙ Document COVID status at the regional level over time ∙ Document COVID status at the clinic level over time ∙ Measure participant-level impact of COVID over time ∙ Consider evaluation of participant outcomes at longer (shorter) lags to determine potential impacts on response trajectories	∙ Consider effect modification with proximal measures of COVID impact (patient level, clinic level) ∙ Describe longitudinal trajectories in patient outcomes across treatment groups and across COVID time periods
Setting	∙ Document the research setting (modality) for both intervention delivery and participant assessment	∙ Describe differences in intervention, comparison, and concomitant care across specific research settings ∙ Describe differences in outcomes across research settings

### Reported benefits

Despite the complex challenges brought by COVID-19, many trial teams described some benefits as a result of the pandemic. Notable benefits included sites and staff showing adaptability, staff coming closer together to overcome challenges, the ability to reach a wider population through virtual intervention delivery, the ability to study virtual intervention delivery, and a renewed commitment to the trial from a partner organization. ACP PEACE mentioned innovations to their intervention, including texting patients a video to view before their clinic visits. In Nudge, the study intervention was able to be used to send important information about COVID-19 directly to participants.

### Planning/next steps

Most trial teams indicated they were moving forward with their revised study plans and working with their Institutional Review Boards and Data and Safety Monitoring Boards to gain approval to proceed. The need for frequent communication with stakeholders was a theme as trials began making progress while simultaneously anticipating future issues that might arise due to COVID-19. Because of this, trial teams emphasized the need for continued flexibility. The BackInAction trial will be working with the Biostatistics and Study Design Core to determine whether refinements in their analytic approach may be warranted. The Collaboratory Coordinating Center and Core Working Groups will continue to stay in close contact with trial teams and offer support as they navigate issues related to COVID-19.

## Discussion

While the COVID-19 pandemic has resulted in numerous scientific milestones, it has also produced major disruptions to the research enterprise. In this qualitative study, we characterized the impact of COVID-19 on research activities of 14 pragmatic clinical trials in the NIH Health Care Systems Research Collaboratory. Our main findings include the following: (1) project teams primarily described impacts related to healthcare operations and social distancing, each of which resulted in project delays; (2) for many projects, the pandemic necessitated changes to the intervention, implementation plans, and data collection strategies; (3) the process for making these changes was driven primarily by existing stakeholders, including funders, the project Coordinating Center, health systems, and the trial team; (4) key benefits of pandemic-related modifications included expanded outreach capabilities and use of virtual interventions; and (5) all trial teams reported successful adaptation to pandemic-related disruptions.

While the pandemic affected ongoing trials in numerous ways, the most consistently reported impacts were delays in activities due to changes in healthcare operations and social distancing. In late spring 2020, many US-based academic research activities were paused, particularly those unrelated to COVID-19 or otherwise deemed “non-essential” [10]. As the pandemic extended into the summer, many research activities resumed under modified conditions as study teams prepared for longer-term disruptions associated with COVID-19 [11]. As with other clinical research, the Collaboratory’s Demonstration Projects reported making preparations to accommodate this extended disruption, including staff adaptation to remote work or re-allocation to COVID-19-related research. However, given their close connection with the healthcare system, pragmatic trials in the Collaboratory were affected by more persistent changes to the healthcare environment itself, including delays due to widespread shifts to virtual healthcare and suspension of some healthcare services. Given that some of these shifts are likely to become permanent [12], lessons learned by pragmatic trial teams, such as the support needed to adapt in-person interventions to virtual interventions, will be informative for future pragmatic research in the post-COVID era.

Despite major changes to the research environment and multiple delays due to system-wide research pauses and staffing changes, most trial teams were able to adapt their interventions and/or data collection strategies to continue making safe progress toward research aims in the new environment. These changes ranged from major (restarting the trial) to minor (monitoring data missingness), but most required engagement of stakeholders outside of the immediate trial teams. Several trials were able to successfully adapt in-person interventions for virtual delivery and collect qualitative data through virtual focus groups. Central to most successful adaptations was support from stakeholders at the health system and funder level, as well as direct consultation with Collaboratory Cores when specific guidance was needed.

While most trial teams acknowledged negative impacts of the pandemic on research activities, the degree of negative impact varied by trial. Trials that were able to assume that any changes in outcome rates due to COVID-19 were non-differential by treatment arm, or that patterns of diagnosis were consistent with the pre-COVID period, reported the fewest impacts. Some trial teams reported benefits as a result of changes made, including greater inclusiveness when using virtual interventions. Virtual trials have received increasing attention as a way to enhance diversity and decrease financial and geographic barriers to participation [13]. Reducing these barriers can be especially useful for populations with transportation limitations or physical impairments. However, virtual interventions and/or data collection may be unfamiliar to populations that are less technologically savvy, and require additional support as a result. Additional research quantifying the impacts of virtual intervention delivery and data collection on study population characteristics, data completeness, and participant engagement is needed.

Finally, to guide future work in this space, we summarized several considerations related to measurement and analysis of COVID-19 impacts within each PICOTS domain. While this framework can be readily applied to characterize and mitigate COVID-related impacts, it may also be relevant for other disruptions that meaningfully impact research conduct and reporting within dynamic or unpredictable environments. Consensus-based reporting guidelines, such as the CONSERVE statement [14], may additionally improve transparency when describing important modifications to trials made as a result of the pandemic.

### Limitations

There are several limitations to our study worth noting. First, while pragmatic trials included in our survey sample covered a broad range of therapeutic areas and intervention types, they are a selected subset supported through the Collaboratory and their experiences may not be generalizable to those of other pragmatic trials. Second, because our survey was administered in June 2020, we were unable to examine longitudinal changes in trial impacts as the pandemic progressed. Finally, while our survey included questions related to multiple potential impacts, it is possible the pandemic impacted trial conduct in ways not captured through our survey or discussions with project teams. Future research examining the pandemic’s impact on pragmatic trials in different settings and over the course of the pandemic is needed.

## Conclusion

The COVID-19 pandemic has disrupted pragmatic clinical research activities in multiple ways. Key impacts on the conduct of ongoing pragmatic clinical trials included changes to healthcare operations and social distancing-related effects, many of which resulted in modifications to interventions and changes to data collection strategies. Investigators also described that, while research priorities are often seen as lower priority than clinical activities for many health systems, the pandemic accentuated clinical priorities even further and required greater persistence from the study teams. Identifying and engaging appropriate stakeholders at the system and study team level were critical to designing and implementing COVID-19-related trial modifications. In addition to the many challenges, several benefits were identified, including expanded outreach capabilities and ability to study virtual intervention delivery. All trial teams reported moving forward with revised study plans and emphasized the importance of stakeholder engagement as they continue to make progress on study objectives.

## Supplementary Information

**Additional file 1.** Questionnaire: Effect of COVID-19 on NIH Collaboratory Embedded Pragmatic Clinical Trials.

## Data Availability

Data and materials for this manuscript will be made available upon reasonable request.

## Abbreviations

ACP: Advance care planning
Collaboratory: NIH Health Care Systems Research Collaboratory
HTE: Heterogeneity of treatment effect
PICOTS: Population, Intervention, Comparison, Outcome, Timing, and Setting
NIH: National Institutes of Health
