# Supplementary material for: The impact of COVID-19 on pragmatic clinical trials: lessons learned from the NIH Health Care Systems Research Collaboratory
Source: Trials. 2022 May 21;23:424. doi: 10.1186/s13063-022-06385-8 (PMC9123729; doi:10.1186/s13063-022-06385-8)
Supplement: Supplementary file 1 — Additional file 1. Questionnaire: Effect of COVID-19 on NIH Collaboratory Embedded Pragmatic Clinical Trials. [file 13063_2022_6385_MOESM1_ESM.docx]

**Supplementary Material**

**Questionnaire: Effect of COVID-19 on NIH Collaboratory Embedded Pragmatic Clinical Trials**

*All responses are free text*

1. How has COVID-19 impacted your trial?
2. What challenges or barriers have you encountered in your trial because of COVID-19?
3. What solutions are you trying to address these challenges or barriers?
4. What, if any, benefits have resulted to your trial because of COVID-19?
5. Which key individuals or stakeholders have you engaged due to COVID-19?
6. Are you using any new measures because of COVID-19?
7. What changes or adaptations have you made to your intervention(s) as of result of COVID-19?
8. What changes have you made to your implementation plans because of COVID-19?
9. What COVID-19-related changes have you noticed at the local, regional, or national level that you think may impact your trial?
10. What are your next steps going forward to address the impact of COVID-19 on your trial?
11. Would you like to mention anything else not covered previously?
